# Two new species of the genus *Ancistrocerus* Wesmael (Hymenoptera, Vespidae, Eumeninae) from China, with a key to the Oriental species

**DOI:** 10.3897/zookeys.303.4922

**Published:** 2013-05-21

**Authors:** Ju You, Bin Chen, Ting-jing Li

**Affiliations:** 1 Institute of Entomology & Molecular Biology, College of Life Sciences, Chongqing Normal University, Chongqing 401331, China

**Keywords:** Hymenoptera, Vespidae, Eumeninae, *Ancistrocerus*, new species, China

## Abstract

Two new species, namely *Ancistrocerus transpunctatus* You and Li, **sp. n.** and *Ancistrocerus deqinensis* You and Li, **sp. n.** are described and illustrated from Yunnan, China. A key to the Oriental species of the genus *Ancistrocerus* is provided.

## Introduction

The key characters of the genus *Ancistrocerus* characterized as follows: pronotal carina weak dorsally (in some species obliterated) but strongly developed laterally; width of metasomal tergum I much greater than length, basally with a transverse carina; tergum II basally with a transverse sulcus, and with longitudinal keels on the bottom of the sulcus (Kim and Yamane 2009). Up to now, 114 species and 42 subspecies were recorded worldwide, including 58 species and 17 subspecies from the Palearctic Region (Blüthgen 1954; Borsato 2006; Cameron 1911; Giordani Soika 1964a; Gusenleitner 1977, 1995; Kim and Yoon 1995; Kim and Yamane 2009; Pekkarinen and Hulden 1991; van der Vecht and Fischer 1972; Yamane 1990), 16 species from the Oriental Region (Bingham 1897; Cameron 1900, 1908; Giordani Soika 1964b, 1976, 1991, 1994; Gusenleitner 1997, 2010; Kim and Yamane 2009; Li 1982, 1985; Meade–Waldo 1910a, 1910b, 1913; Yamane and Gusenleitner 1993), 22 species and 12 subspecies from the Ethiopian Region (Carpenter et al. 2009), 19 species and 12 subspecies from the Nearctic Region (Bequaert 1925, 1944; Buck et al. 2008; Cameron 1908), and 12 species from the Neotropical Region (Bequaert 1925; Carpenter and Garcete–Barrett 2002; Carpenter and Genaro 2011). Twenty–six species and two subspecies of this genus were already recorded from China (Bingham 1897; Giordani Soika 1964b, 1970, 1976, 1991; Gusenleitner 1993; Meade–Waldo 1910a, 1910b; Yamane and Gusenleitner 1993). In the study of *Ancistrocerus* from China, additional two new species are found from Yunnan. In the present paper, these two new species are described and illustrated in detail, along with a key to the Oriental species of *Ancistrocerus*. The key is produced based on both the examination of specimens and the characters extracted from literatures. The sources of information are listed in the key.

## Materials and methods

The examined specimens were deposited in the Institute of Entomology and Molecular Biology, Chongqing Normal University, Chongqing, China (CQNU); Department of Entomology, Yunnan Agricultural University, Yunnan, China (YNAU). Morphological terminology follows Carpenter and Cumming (1985) and Yamane (1990). Descriptions and measurements were performed under a stereomicroscope (Nikon SMZ1500), and photomicrographs were taken with a stereomicroscope (LEICA EZ4HD) attached to a computer using Leica Application Suite version 2.1.0 software. Body length was measured from the anterior margin of head to the posterior margin of metasomal tergum II.

## Taxonomy

### Ancistrocerus

Wesmael, 1836

Ancistrocerus Wesmael, 1836: 45; Li 1985: 118; Carpenter 1986: 64; van der Vecht and Fischer 1972: 108; Yamane 1990: 98; Kim and Yamane 2009: 31.

#### Type species.

*Vespa parietum* Linnaeus, designated by Giraud 1879.

### Ancistrocerus transpunctatus

You & Li sp. n.

urn:lsid:zoobank.org:act:7765F0E3-CB31-47E2-97A5-FC64DB58A64D

http://species-id.net/wiki/Ancistrocerus_transpunctatus

Figs 1–6

#### Material examined.

Holotype. ♂, China, Yunnan, Diqing, Weixi County, Tacheng Town, 27°36.22'N, 99°24.29'E, 2017 m, 16. VII. 2011, Tingjing Li, No. 201107166 (CQNU). Paratypes. 1♂, China, Yunnan, Diqing, Weixi County, Tacheng Town, 27°36.22'N, 99°24.29'E, 2017 m, 16. VII. 2011, Tingjing Li, No. 201107167 (CQNU); 2♂♂, China, Yunnan, Baoshan City, Tengchong County, Jietou Village, Datang, 25°25.40'N, 98°39.27'E, 1597 m, 13. IV. 2006, Li Ma (YNAU).

#### Description.

**Figures 1–6.**
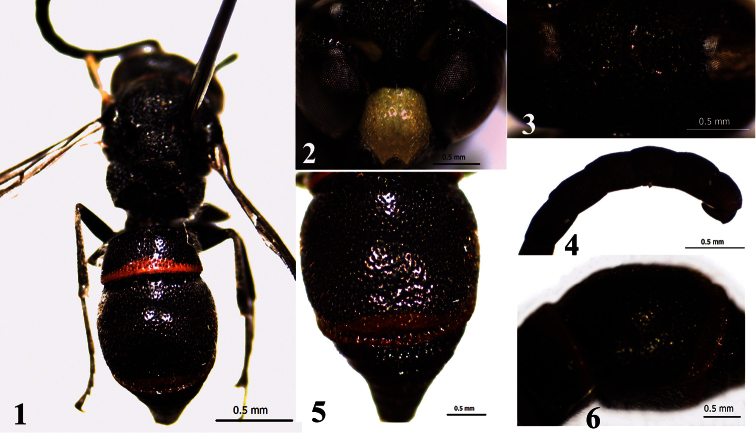
Male of *Ancistrocerus transpunctatus* You,sp. n. **1** general habitus **2** frons and clypeus **3** head in dorsal view **4** antennal articles **5** apical margin of metasomal tergum II **6** metasomal sternum II in profile.

Male: Body length 7.5–8.0 mm (Fig. 1), forewing length 6.0–6.5 mm. Black; the following parts are yellow: clypeus, a spot between antennal socket and eye, labrum, almost mandible, a spot on tempora, outer face of fore tibia, a spot on apex of mid tibia; the following parts are dark ferruginous: antennal article XI, an anterior band on pronotum, apical bands on metasomal terga I–II and sternum II.

Head. Densely covered with long setae, as long as the distance between the posterior ocelli; vertex with dense and coarse punctures, punctures almost connected (Fig. 3); clypeus with sparse punctures (Fig. 2), length of clypeus slightly longer than width, apical emargination slightly shallow, shallower than semicircular, apical teeth somewhat acute; antennal scape with sparse and small punctures, antennal article XIII folded backward, reaching the base of article XI (Fig. 4).

Mesosoma. Setae on mesosoma slightly sparser and shorter than those on the head; pronotal carina weaker on dorsum, but acutely produced in lateral corner; mesopleuron with large and irregular punctures; pronotum and mesonotum with dense and coarse punctures, smaller than those on mesopleuron; tegula slightly smooth and shining, with fine punctures; scutellum flat, metanotum convex, punctures on scutellum and metanotum similar to those on pronotum and mesonotum; marginal and median carinae of propodeum developed, apical convavity of propodeum densely with striae; femora with short white pubescence.

Metasoma. Setae on metasomal tergum I as long as those on mesosoma, but much sparser; length of setae on terga II–VI less than 1/2 times those on tergum I; width of tergum I 2.3 times length, transverse carina well developed and with a narrow and shallow median notch; width of tergum II: length = 2.3: 2.4, the bottom of basal sulcus with longitudinal keels, punctures on metasomal tergum II distinctly weaker than those on tergum I, apical margin of tergum II with a transverse row of big punctures (Fig. 5); metasomal terga III–IV reticulate, densely covered with large punctures; punctures on terga V–VI smaller and weaker than those on terga III–IV; metasomal sternum II deeply truncated behind the basal sulcus, straight and distinctly angled near the base in profile (Fig. 6); sterna II–VI with sparse and small punctures.

Female. Unknown.

#### Remarks.

The species is similar to *Ancistrocerus antoni* (Cameron, 1900) from India, in body coloration with similar spots, pronotal carina acutely produced into lateral corner, and shape of the tegula. But it can be distinguished from the relatedspecies and other members of the genus with the following characters: apical margin of metasomal tergum II with a transverse row of big punctures, forming a transverse furrow (Fig. 5), terga III–IV reticulate, densely with large punctures.

#### Distribution.

China (Yunnan).

#### Etymology.

It is named after its metasomal tergum II with a transverse row of big punctures.

### Ancistrocerus deqinensis

You & Li sp. n.

urn:lsid:zoobank.org:act:BD1846E2-394C-4E4E-9EC0-1C9CDD811276

http://species-id.net/wiki/Ancistrocerus_deqinensis

Figs 7–14

#### Material examined.

Holotype.♂, China, Yunnan, Diqing, Deqin County, 28°29.03'N, 98°54.63'E, 3467 m, 19. VII. 2011, Tingjing Li, No. 201107191 (CQNU). Paratypes. 4♂♂,the same data as holotype, No. 201107192–201107195 (CQNU).

#### Description.

**Figures 7–14.**
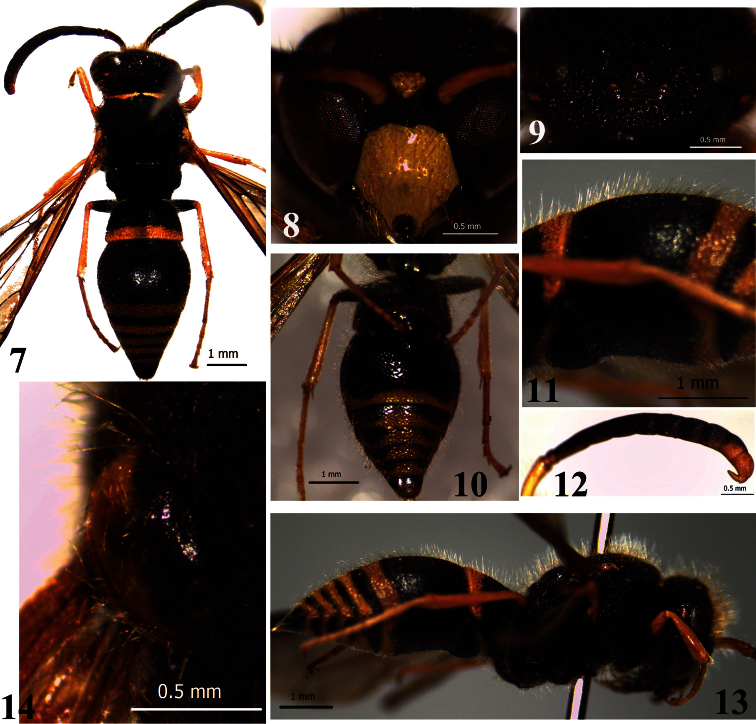
Male of *Ancistrocerus deqinensis* You,sp. n. **7** general habitus **8** frons and clypeus **9** head in dorsal view **10** metasoma in ventral view **11** metasomal sternum II in profile **12** antennal articles **13** general habitus in profile **14** tegula.

Male: Body length 7.0–7.8 mm (Fig. 7), forewing length 7.5–8.0 mm. Black; the following parts are yellow: a lower frontal spot, clypeus, a spot between antennal socket and eye, almost mandible; the parts are bright ferruginous: labrum, antennae ventrally, a spot on tempora, an anterior band on pronotum, outside half of tegula (Fig. 14), apical bands on metasomal terga I–VI and sterna I–VI, a median spot on sternum VII (Fig. 10), and apex of femora to tarsi V in all legs.

Head. Densely covered with long setae, setae distinctly longer than the distance between the posterior ocelli; vertex with dense and coarse punctures, interspaces between punctures ridge–like (Fig. 9); width of clypeus equal to or slightly longer than length, clypeus moderately emarginate, almost semicircular, apically with acute teeth, sparse punctures and long setae (Fig. 8); antennal scape with sparse and small punctures, dense and long setae; antennal article XIII folded backward, reaching nearly the base of article XI (Fig. 12).

Mesosoma. Densely covered with long setae, similar to those on head; pronotal carina weaker in dorsum, but acutely produced in lateral corner. Mesopleuron reticulate, with large and irregular punctures; pronotum and mesonotum with dense and coarse punctures, smaller than those on mesopleuron; tegula with sparse punctures and long setae (Fig. 14); scutellum flat, metanotum convex, punctures on scutellum and metanotum similar to those on pronotum and mesonotum; marginal and median carinae of propodeum well developed, convavity of propodeum with striae; femora with dense long setae and sparse small punctures.

Metasoma. Densely covered with long setae, as long as the distance between the posterior ocelli; width of tergum I slightly less than 2 times length, with somewhat dense large punctures (interspaces smaller than punctures), transverse carina well developed, with a wide and deep median notch; apical bands on metasomal sterna I–VI complete; width of tergum II: length = 2.4: 2.0, the bottom of basal sulcus with longitudinal keels; punctures on terga II–VI much smaller than those on tergum I; sternum II basally with transverse uniform sulcus, not truncate behind sulcus, in profile somewhat concave (Fig. 11), punctures on sterna II–VI much sparser than those on tergum II.

Female. Unknown.

#### Distribution.

China (Yunnan).

#### Remarks.

The species is similar to *Ancistrocerus parietum* (Cameron, 1900) from Europe to northeast of China and North America, in the shape of the clypeus, punctures on the mesosoma, transverse carina of tergum I well developed and with a wide and deep median notch. However, it can be distinguished from similarspecies and other members of the genus with the following characters: body markings bright ferruginous, tegula with sparse punctures and long setae (Fig. 14), terga II–VI with dense long setae (Fig. 13), apical bands on metasomal sterna I–VI complete and sternum VII with a medial spot (Fig. 10).

#### Etymology.

It is named after the type locality of the species, Deqin County in Yunnan Province of China.

##### Key to the Oriental species of *Ancistrocerus*

**Table:** 

1	Setae on frons and vertex distinctly longer than the distance between the posterior ocelli; metasomal terga II–VI with dense long setae	2
–	Setae on frons and vertex as long as or shorter than the distance between the posterior ocelli; metasomal terga II–VI with very sparser and shorter setae	3
2	In male clypeus shallowly emarginate, almost semicircular (Fig. 8)	*Ancistrocerus deqinensis* sp. n.
–	In male clypeus deeply emarginate, distinctly deeper than semicircular (Gusenleitner 2010)	*Ancistrocerus extremus* Gusenleitner
3	Metasomal sternum II behind basal sulcus with a somewhat deep truncation, nearly as high as length of median part of basal sulcus, anterior truncate slope of sternum II distinguished from posterior horizontal part in profile	4
–	Metasomal sternum II behind basal sulcus with shallow truncation, less than half length of median part of basal sulcus, or almost lack of truncation, sternum II smoothly convex in profile	12
4	Length of clypeus longer than width	5
–	Length of clypeus shorter than width	7
5	Apical margin of metasomal tergum II with a transverse row of big punctures, forming a transverse furrow (Fig. 5)	*Ancistrocerus transpunctatus* sp. n.
–	Apical margin of metasomal tergum II normal, without a transverse row of big punctures	6
6	Antennal scape with sparse small punctures, interspaces always larger than punctures	*Ancistrocerus antoni* (Cameron)
–	Antennal scape with dense large punctures, interspaces equal to or smaller than punctures (Giordani Soika 1976)	*Ancistrocerus aureovillosus* Giordani Soika
7	In profile, border rounded between anterior slope and posterior horizontal part of metasomal sternum II	8
–	In profile, border angled between anterior slope and posterior horizontal part of metasomal sternum II	11
8	Clypeus with dense punctures, interspaces smaller than punctures	9
–	Clypeus with sparse punctures, interspaces larger than punctures	10
9	Propodeal dorsum with distinct punctures and shining (Giordani Soika 1994)	*Ancistrocerus handschini* (Schulthess)
–	Propodeal dorsum with indistinct punctures and dull (Giordani Soika 1994)	*Ancistrocerus borneanus* Giordani Soika
10	Metasomal terga III–V with apical bands (Gusenleitner 1996)	*Ancistrocerus rufoluteus* Gusenleitner
–	Metasomal terga III–V without apical bands (Yamane and Gusenleitner 1993)	*Ancistrocerus montuosus* Gusenleitner
11	Metasomal terga III–IV with apical bands; female without a spot between antennal socket and eye (Kim and Yamane 2009)	*Ancistrocerus nigricornis* (Curtis)
–	Metasomal terga III–IV without apical bands; female with a yellow spot between antennal socket and eye (Yamane and Gusenleitner 1993)	*Ancistrocerus terayamai* Yamane
12	Mesosoma and metasoma with ivory–white spots (Meade-Waldo 1910)	*Ancistrocerus hirsutus hirsutus* (Meade–Waldo)
–	Mesosoma and metasoma with yellow or ferruginous spots	13
13	Metasomal tergum III with apical band	14
–	Metasomal tergum III without apical band	15
14	Concavity of propodeum laterally sculptured, and apically dull (Gusenleitner 1996)	*Ancistrocerus xanthozonus* (Curtis)
–	Concavity of propodeum not laterally sculptured, and apically shining	*Ancistrocerus antilopeantilope* (Panzer)
15	Metasomal tergum I with sparse punctures, interspaces equal to or larger than punctures	16
–	Metasomal tergum I with dense punctures, interspaces always smaller than punctures	17
16	Metanotum convex; propodeum with well developed superior ridges (Kim and Yamane 2009)	*Ancistrocerus philippinus* Giordani Soika
–	Metanotum not convex; propodeum with weak superior ridges (Giordani Soika 1971)	*Ancistrocerus sikhimensis* (Bingham)
17	Head and mesosoma with large punctures; mandible with a yellow spot; antennal scape always with a yellow spot; clypeus in female basally with two yellow spots (Giordani Soika 1991)	*Ancistrocerus arcanus* Giordani Soika
–	Head and mesosoma with small punctures; mandible and antennal scape in male, and clypeus in female, black (Giordani Soika 1991)	*Ancistrocerus waltoni* (Meade–Waldo)

## Supplementary Material

XML Treatment for Ancistrocerus

XML Treatment for Ancistrocerus transpunctatus

XML Treatment for Ancistrocerus deqinensis
